# Supplementary material for: Prevalence of and risk factors for hepatitis B and C viral co-infections in HIV infected children in Lagos, Nigeria
Source: PLoS One. 2020 Dec 10;15(12):e0243656. doi: 10.1371/journal.pone.0243656 (PMC7728231; doi:10.1371/journal.pone.0243656)
Supplement: S1 Appendix — (DOCX) [file pone.0243656.s001.docx]

**APPENDIX 1**

**QUESTIONNAIRE**

Please fill in the appropriate information or tick the appropriate answer.

All information provided will be treated with strict confidentiality.

**SECTION A: Socio-demographic factors**

1. Q1 Serial number……………..
2. Q2 Gender male [ ] female [ ]
3. Q3 Age
4. Q4 Religion Christians

Muslims

African Traditional Religion

Others state

1. Q5 Ethnic group……………………………………………………………
2. Q6 Residential address…………………………………………………………
3. Q7 No of members in the family ……………………………………..
4. Q8 No of rooms ……………………………………………………….
5. Phone number …………………………………………………….
6. Q9 Educational status of father
7. No formal education
8. Primary
   - 1. Completed
     2. Not completed
9. Secondary
   - 1. Completed
     2. Not completed
10. Post-secondary
11. Others (specify)
12. Q10 Educational status of mother
13. No formal education
14. Primary
    - 1. Completed
      2. Not completed
15. Secondary
    - 1. Completed
      2. Not completed
16. Post-secondary
17. Others (specify)
18. Q11 Fathers occupation (state)………………………………………….
19. Q12 Mothers occupation (state)…………………………………………..

**SECTION B: Clinical features**

1. Q13 Jaundice [Yes] [No]
2. Q14 Weight ……………………..
3. Q15 Height ………………………
4. Q16 Pallor [Yes] [No]
5. Q17 Temperature ^0^c …………………………………………..
6. Q18 Itching [Yes] [NO]
7. Q19 Splenomegaly [YES] [NO]
8. Q20 Hepatomegaly [YES] [NO]
9. Q21 Dark coloured urine [TES] [NO]
10. Q22 Body weakness [YES] [NO]
11. Q23 Clinical stage of paediatric HIV in subjects …………………….

**SECTION C: Blood Transfusion History**

1. Q24 Have you been previously hospitalized? [YES] [No]

Q24a If yes, why (with date) …………………………………………………………………………

Q24b How many times have you been hospitalized in the past?………………………………………………..

1. Q25 Have you been previously transfused with blood? [YES] [No]

Q25a If yes, number of times transfused ……..………………………………………...

Q25b Was the blood screened for HIV [YES [NO [Don’t know

Q25c Was the blood screened for HBsAg[YES [NO [Don’t know

Q25d Was the blood screened for anti HCV [YES [NO [Don’t know

Q26 Where was the transfusion done?

1. Teaching Hospital
2. General Hospital
3. Private hospital
4. Laboratory
5. Others (specify)

**SECTION D: Other risk factors**

1. Q27 Do you have incision marks / Tatoos? [YES] [No]

Q27a Instrument used (if Yes)……………………………………………………………………

Q27b Have you taken hepatitis B vaccination in the past? [YES] [No]

Q27c Where was it given?……………………………………………..

Q27d How many doses?………………………………………………..

Q27e At what age was it taken? …………………………………………

Q27f Immunization card sighted [YES] [No]

1. Q28 Do you practise sharing of needles? [YES] [No]
2. Q29 Do you practise sharing of tooth brush? [YES] [No]
   1. Q29a If Yes, with whom? ………………………..
3. Q30 Have you had surgical operations in the past? [YES] [No]
4. Q31 Have you experienced genital circumcision in the past?[YES] [No]
5. Q32Have you had sexual exposure in the past? [YES] [No]
6. Q33 Have you had jaundice in the past? [Yes [No

SECTION E: RESULT

1. Q34 HBsAg POSITIVE NEGATIVE
2. Q35 anti HBC POSITIVE NEGATIVE
3. Q36 anti HCV POSITIVE NEGATIVE
4. Q37 Socio-Economic Status: I-II…. Upper

III…. Middle

IV-V…. Lower
