# Supplementary material for: Prevalence of and risk factors for hepatitis B and C viral co-infections in HIV infected children in Lagos, Nigeria
Source: PLoS One. 2020 Dec 10;15(12):e0243656. doi: 10.1371/journal.pone.0243656 (PMC7728231; doi:10.1371/journal.pone.0243656)
Supplement: Attachment — Submitted filename: Response to Reviewers.odt [file pone.0243656.s003.odt]

Response to Reviewers
2. In your Methods section, please provide additional information about the participant recruitment method and the demographic details of your participants. Please ensure you have provided sufficient details to replicate the analyses such as:
a) the recruitment date range (month and year),
b) a description of any inclusion/exclusion criteria that were applied to participant recruitment,
c) a statement as to whether your sample can be considered representative of a larger population, and
d) a description of how participants were recruited.
Authors response
Recruitment of study participants was done at the HIV clinic, well-baby and the Paediatric outpatient clinics using the systematic sampling method.
The demographic details are provided as follows; 
Age, gender, ethnicity and socioeconomic status (details are shown in page 8 of the manuscript)

The recruitment date range (month and year), is already provided in the manuscript (August 2017 to December 2017).

STUDY CRITERIA
Inclusion criteria for subjects (HIV infected)
Children aged between 2 months to 13 years who are HIV positive following the HIV screening algorithm.
Children whose parents/guardians gave informed consent to participate in the study; (and assent obtained for children 7years and above).
Those that completed the administered questionnaire properly.

Exclusion criteria for subjects (HIV infected)
Children whose parent(s) / guardian refused or withdrew their consent.
Those with acute febrile illness, seizure disorders and cerebral palsy.

Inclusion criteria for HIV naive (healthy controls)
Children aged between 2 months to 13 years with no evidence of HIV infection on a rapid screening test.
Children whose parents/guardians gave informed consent to participate in the study; (and assent obtained for children 7 years and above).
Participants who completed the administered questionnaire properly.

Exclusion criteria for HIV naive (healthy controls)
Children whose parent(s) / guardians refused or withdrew their consent.
Children with chronic diseases such as confirmed sickle cell anaemia, confirmed chronic liver disease, cerebral palsy and seizure disorders.

Yes, the sample can be considered representative of a larger population. The study sample size was determined using appropriate methodology. 
The minimum sample size was determined using the formula for calculating sample size for the comparison of two independent proportions. (28)
n per group = 2 (Zα + Zβ) 2P (1─ P)	
(Po─ P1)2
Where		
Zα = the standard normal deviate of α at 95% confidence interval, (the probability of making a type 1 error) = 1.96
Zβ = the standard normal deviate of β (the power) is set at 90% = 1.28
 Po = the prevalence of hepatitis B co-infection among HIV-infected children
A prevalence of 10% was used based on the work by Durowaye et al (29)
P1 = the prevalence of hepatitis B in children;
A prevalence of 0.5% was used based on the work by Okonko et al (30)
P is the arithmetic average of the two proportions Po+ P1
2

Patients were recruited using the systematic sampling method.  
From the records in the HIV paediatric clinic of LUTH, the average monthly attendance of children was about 100 (with the clinic running once a week); therefore, an assumption of average weekly attendance of 25 children was made. A list of all registered patients for each clinic attendance was obtained from the recording officer. The 1st participant was randomly selected from the list and subsequently, every 4th number on the list was selected. An average of 12 children whose parents gave permission and/ assents was selected every clinic day from among the attendees, and this process continued until the minimum required sample size determined by appropriate sample size and representative of the larger population was achieved. For every 4th name that declined the 5th name was chosen.
Recruitment of study participants was done at the paediatric HIV clinic of LUTH over five months (August 2017 to December 2017). The researcher on each clinic day addressed all the parents/ guardian and the patients present where details of the study were communicated to them. Consent and assent forms were given to parents or guardians and to patient aged 7 to 13 years who satisfied the inclusion criteria. Consenting parents or guardian and children 7-13 years were subsequently recruited into the study.
The same method was used to recruit healthy controls from the well-baby clinic and paediatric outpatient unit. These children were age and sex-matched with the subjects.
3.Genital circumcision is defined as the removal of the foreskin from the penis in this manuscript, as seen on page 8
4. Please provide the name and catalogue number of the anti-HCV Version 4.0 Immunoassay.
Response: Version 4.0 Enzyme Immunoassay; DIA.PRO Diagnostic Bioprobes Srl, Italy.) HCV Ab (DIA.PRO), Lot C11T12/5.
Comments to the Author
5.Review Comments to the Author
Reviewer #1: 1. When describing the prevalence of HIV/HBV and HIV/HBV respectively, especially in the Introduction when describing the published literature, please be clearer and describe the range prevalence for each of the viral co-infections. In the current form, it is rather unclear what the authors are referring to in their ranges of prevalence.
Response: The prevalence of HIV/HBV and HIV/HCV in the introduction has been addressed as seen in pages 4 and 5.
2. In the discussion, more can and should be said about whether there are variations in the rate of Hepatitis B vaccination between the different regions in Nigeria in which the prevalence of HIV/HBV has been studied, to account for the differences reported.
Response: There are variations in the rate of Hepatitis B vaccination between the different regions in Nigeria which has been addressed in the discussion as seen in pages 19 and 20.
3. The discussion is rather confusingly structured. Would recommend that the authors reframe it speaking of each of the viral hepatitides in sequence, without the current structure which shifts between HBV and HCV, for ease of understanding.
Response: Each viral hepatitis has been discussed in sequence in the discussion as seen in pages 18 to 22. 
4. A thorough proofreading and rewriting were needed to make it easier to read and understand would also help in the overall quality of the manuscript.
Response: It has been addressed accordingly.
Please upload a copy of supporting information Figure S1 and tables S1-S5 which you refer to in your text on page 30
Response: Figure 1 already uploaded as a supporting file.
For the tables, the guideline for submission says that ‘do not submit tables in a separate file(s). Place each table in your manuscript file directly after the paragraph in which it is first cited. However, a separate file that contains Tables 1-5 has been uploaded as requested.
2. Please add the definition of Genital circumcision to your methods section.
Response: The definition has been added to the method section on page 8.


 
